# Association study between the gibberellic acid insensitive gene and leaf length in a *Lolium perenne *L. synthetic variety

**DOI:** 10.1186/1471-2229-11-183

**Published:** 2011-12-28

**Authors:** Jérôme Auzanneau, Christian Huyghe, Abraham J Escobar-Gutiérrez, Bernadette Julier, François Gastal, Philippe Barre

**Affiliations:** 1 INRA, UR4, Unité de Recherche Pluridisciplinaire Prairies et Plantes Fourragères, Le Chêne, RD 150, 86600 Lusignan, France

## Abstract

**Background:**

Association studies are of great interest to identify genes explaining trait variation since they deal with more than just a few alleles like classical QTL analyses. They are usually performed using collections representing a wide range of variability but which could present a genetic substructure. The aim of this paper is to demonstrate that association studies can be performed using synthetic varieties obtained after several panmictic generations. This demonstration is based on an example of association between the gibberellic acid insensitive gene (GAI) polymorphism and leaf length polymorphism in 'Herbie', a synthetic variety of perennial ryegrass.

**Methods:**

Leaf growth parameters, consisted of leaf length, maximum leaf elongation rate (LERmax) and leaf elongation duration (LED), were evaluated in spring and autumn on 216 plants of Herbie with three replicates. For each plant, a sequence of 370 bp in GAI was analysed for polymorphism.

**Results:**

Genetic effect was highly significant for all traits. Broad sense heritabilities were higher for leaf length and LERmax with about 0.7 in each period and 0.5 considering both periods than for LED with about 0.4 in each period and 0.3 considering both periods. GAI was highly polymorphic with an average of 12 bp between two consecutive SNPs and 39 haplotypes in which 9 were more frequent. Linkage disequilibrium declined rapidly with distance with r ^2 ^values lower than 0.2 beyond 150 bp. Sequence polymorphism of GAI explained 8-14% of leaf growth parameter variation. A single SNP explained 4% of the phenotypic variance of leaf length in both periods which represents a difference of 33 mm on an average of 300 mm.

**Conclusions:**

Synthetic varieties in which linkage disequilibrium declines rapidly with distance are suitable for association studies using the "candidate gene" approach. GAI polymorphism was found to be associated with leaf length polymorphism which was more correlated to LERmax than to LED in Herbie. It is a good candidate to explain leaf length variation in other plant material.

## Background

Genetic association studies using accessions of unknown pedigree are increasingly used in plant biology to identify genes explaining variation of traits. Indeed, when compared to quantitative trait loci (QTL) analyses, these studies present the advantage of i) comparing concomitantly several alleles, ii) avoiding laborious population constructions and iii) exploiting the recombination events that have occurred over several generations [1-3]. For association studies, two approaches are possible base on the pattern of linkage disequilibrium (LD) decline [4]. The first one is the candidate gene approach used for populations showing a rapid LD decline. The second one is the whole genome scan approach used for populations showing a slow LD decline [4]. These two approaches have been mainly used for analysing the genetic variability of a species through core collections. However, core collections are often genetically structured, thus leading to the detection of spurious associations between the polymorphism of markers and traits [5-7]. In order to circumvent the detection of spurious associations, different methods of data analysis have been developed that take into account the core collection's substructure [5]. Nevertheless, the use of these methods allows only to study the intra-group variability while leaving the inter-group variability unexploited. Ideally, the best plant material for association studies should be multi-allelic and without any substructure. This is the case in synthetic varieties, obtained after several panmictic multiplication generations, as shown by Auzanneau [8]. However, to our knowledge, there are no previous reports concerning association studies on synthetic plant varieties.

Perennial ryegrass is the most sown forage and turf grass species in temperate climate and it is considered as a model for genomics in forage grasses [9]. In this species, like in all forage grasses, leaf length is an important trait affecting: i) vegetative yield [10-12], ii) intake rate by dairy cows [13], iii) plant survival under light competition conditions [14]. Furthermore, it is a quantitative trait presenting a large variability and a high heritability [15-17].

*GAI *plays an important role in plant growth in several species by acting on the gibberellin signal [18]. Mutants of this gene exist in various species, with dwarf or giant phenotypes. Some dwarf mutants are *OsGAI *in rice [18], *Rht-D1 *in wheat [19] and *gai *in *Arabidopsis *[20,21] and some giant mutants are *SLR1 *in rice [22], *sln *in barley [23,24], and *spy *in *Arabidopsis *[25]. Moreover, *GAI *is mapped on linkage group 3 in rice [26]http://www.ncbi.nlm.nih.gov/entrez/viewer.fcgi?val=13699786&itemID=65&view=gbwithparts and on linkage group 4 in perennial ryegrass [27]. In addition, a QTL of leaf length was found on linkage group 4 in the region of *GAI *[28].

The aim of this paper is to demonstrate that an association study following the candidate gene approach is possible in synthetic varieties. This demonstration is based on an example of association between the gibberellic acid insensitive gene (*GAI*) polymorphism and leaf length polymorphism in 'Herbie', a synthetic variety of perennial ryegrass (*Lolium perenne *L.) which presents no substructure and a short LD decline [8]. Moreover, a core collection of perennial ryegrass was used in order to compare the phenotypic variability observed in 'Herbie' against the variability present within the species.

## Methods

### Plant materials

**Figure 1:**
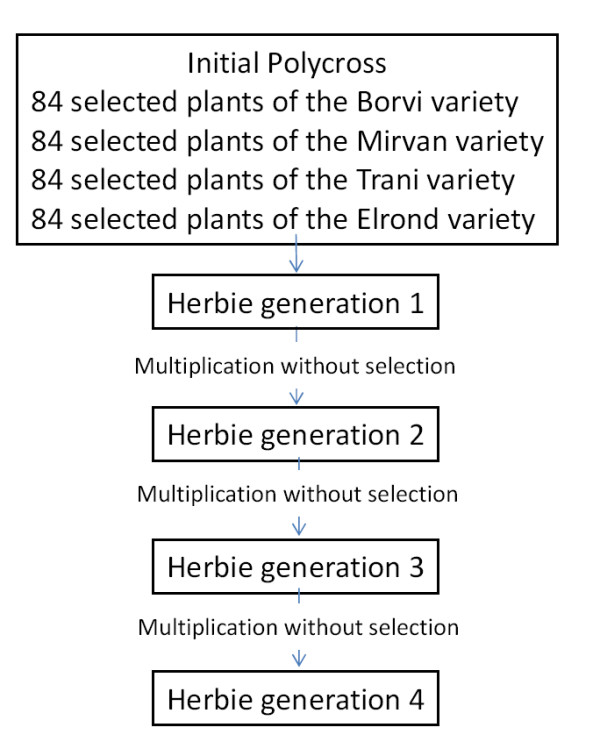
**Description of the creation of the variety 'Herbie'**.

We studied 216 plants of the 'Herbie', a synthetic variety chosen because of its large variability and because LD decreases rapidly [8]. It was registered for the first time in 2000 in France. We used the fourth generation of multiplication after the initial polycross in which 336 parents with 4 different origins were involved (Figure 1; Thieu Pustjens pers. com.). Seeds were sown in summer 2003 in individual pots and stored above 10°C with at least 14 hours of light per day in a greenhouse in order to avoid vernalization until the 24th of February 2004. At this date, three clones per plant of one main tiller were produced for phenotyping in spring and one clone was produced for conservation. This last clone was used to produce three new clones in autumn. We used 100 ecotypes (one plant per population) chosen in an Eurasian core collection in order to maximise the number of geographical origins (François Balfourier, pers. com., Supplementary data). For convenience, we named these 100 ecotypes: core collection (Cc). Seeds of this collection were sown in February 2004 in individual pots and stored above 10°C with at least 14 hours of light per day in a greenhouse until the 30th of September 2004. At this date, three clones per plant of one main tiller were produced.

### Phenotyping

Two experiments with three replicates each were conducted in a greenhouse: the first in spring 2004 with Herbie genotypes and the second in autumn 2004 with Herbie and Cc genotypes. For each plant, one main tiller with its daughter tillers was planted at both periods. Plants were watered and nitrogen supplied in order to avoid any stress. After a growth period of three weeks, plants were cut back. The third and fourth uncut leaves that appeared after this defoliation were measured on one tiller per plant, three times a week from the time of their emergence until they reached maturity. Thermal time was calculated in growing degree days (°Cd) using a simple sum of mean daily air temperature above 0°C taking the day of cutting as a starting point. The spring experiment began on March 17, 2004 and finished on May 12. However, the plants were followed until the end of July in order to confirm their vegetative state by the absence of floral stem. The autumn experiment began on October 21, 2004 and finished on January 12, 2005. During the autumn experiment, the greenhouse was heated to maintain a temperature higher than 10°C.

Final leaf length and leaf growth kinetics of the third and fourth uncut leaves were used as phenotypic character for the association analysis. In order to estimate the parameters of leaf growth kinetics, leaf length measurements for each plant and leaf against thermal time were fitted a function inspired from the Euler integral [12,29,30], such that:

(1) $$Y=Ym\cdot \left(1+\frac{te-t}{te-tm}\right)\cdot {\left(\frac{t-tc}{te-tc}\right)}^{\frac{te-tc}{te-tm}}$$

for *tc *≤ *t ≤ te *and *tc *≤ *tm < te*. For *t > te*, Eq. 1 is reduced to *Y = Ym*.

**Figure 2:**
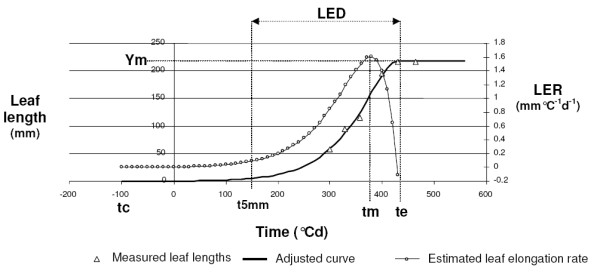
**Example of the Eq.1 function fitted between leaf length and thermal time**. Leaf elongation rate obtained by derivation of the function is shown. Are indicated: the maximum leaf length (Ym), the starting time of elongation with a leaf length of 5 mm (tc), the time when the speed elongation is maximal (tm), the time when the leaf finishes growing (te) and the leaf elongation duration (LED).

*Y *(mm) is the leaf length at any time, *Ym *(mm) is the final leaf length, *tc *the time when leaf growth starts (°Cd), *tm *is the time at which the maximum leaf elongation rate is reached (°Cd) and *te *is the time when leaf growth ends (°Cd). Model fitting was performed by using the NLIN procedure of SAS [31]. Parameters were optimised using the Levenberg-Marquardt iterative method with automatic computation of the analytical partial derivatives [32,33]. Seed values were as followed: *tc *ranged from 0 to 10 increasing by 1; *tm *ranged from 10.001 to 1,000 increasing by 10 and *te *ranged from 150 to 1,200 increasing by 10. For each leaf, *Ym *value was given by its maximum measured length. Only plants with data on both the third and fourth leaves were analysed. An example of the Eq. 1 fitted to data is given in Figure 2.

It follows from Eq. 1 that the leaf elongation duration, *LED*, expressed in thermal time units (°Cd), is given by difference *te *- *tc*. However, because the fitting procedure yielded a few negatives values without physiological meaning for *tc*, and in order to avoid incoherent estimates of *LED*, we numerically estimated the time at which any leaf was 5 mm long, and called this value *tc_5_*. *LED *was then estimated as:

(2) $$LED=te-tc_{5}$$

The first derivative of Eq. 1 gives the leaf elongation rate, *LER*, at any time such that:

(3) $$\frac{dY}{dt}=Ym\cdot \left(\frac{\left(1+\frac{te-t}{te-tm}\right)\cdot {\left(\frac{t-tc}{te-tc}\right)}^{\frac{te-tc}{te-tm}-1}}{te-tm}-\frac{{\left(\frac{t-tc}{te-tc}\right)}^{\frac{te-tc}{te-tm}}}{te-tm}\right)$$

The maximum leaf elongation rate, *LERmax*, for each leaf was numerically obtained from Eq. 3. Summarising, three variables were studied at two growing periods, spring and autumn: final leaf length (*Llength*), leaf elongation duration (*LED*) and maximal leaf elongation rate (*LERmax*).

### DNA extraction

For each Herbie genotype, DNA was extracted from 1 g of young leaf using a cetyltrimethyl ammonium bromide (CTAB) protocol [34,35]. DNA quantity and quality of each sample were assessed on agarose gel.

### Genotyping

Herbie plants were genotyped using one SSR marker per linkage group (LG): B4D7 on LG1, G02-049 on LG2, G07-058 on LG3, G03-10 on LG4, pps397 on LG5, G04-56 on LG6 and G02-004 on LG7 [36,37]. PCR reactions and separation of amplified products were performed as described for SSRs in Barre [28].

**Figure 3:**
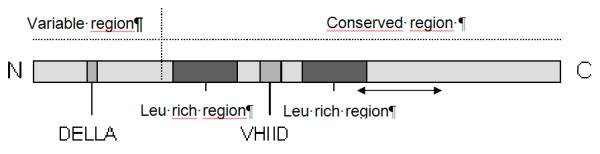
**Representation of GRAS protein structure from Bolle **[38]. DELLA and VHIID protein domains and leucine (Leu) rich regions are indicated. The arrows show the amplified part of GAI studied.

In order to amplify a fragment of the GAI gene in *Lolim perenne *L., degenerated primers were designed on an alignment between *OsGAI *http://www.ncbi.nlm.nih.gov/entrez/viewer.fcgi?val=13699786&itemID=65&view=gbwithparts, *Rht-D1a *(AJ2425311), *SLN-1 *(AF460219) and *DWARF8 *(AJ242530) homologous *GAI *gene in rice, wheat, barley and maize, respectively. A sequence of 370 bp in *GAI *was amplified in the coding region (Figure 3). PCR reactions were set up in 50 μL volumes in 96-well PCR plates. Each PCR reaction was performed with 40 ng of template DNA, 0.4 μM of each primer (5'-GACYTGGAGCCSTTCATGCT-3'; 5'-GTACACCTCSGACATGACCT-3'), 2 mM MgSO_4_, 0.2 mM dNTP, 1 U Platinium Taq DNA polymerase High Fidelity (Invitrogen) and 1X PCR buffer (Invitrogen). The amplifications were performed using a PTC100 thermal cycler with the following program: 10 min at 94°C, followed by 35 cycles of 94°C for 1 min, 58.7°C for 1 min and 68°C for 2 min, a final extension of 10 min at 68°C. PCR product of each sample was purified using QIAquick Multiwell PCR Purification Kit (QIAGEN) and sent to Millegen, Toulouse, France for direct sequencing of PCR products with the forward primer. The direct sequencing of the PCR products allowed to obtain the genotype of each SNP but not the phase between SNPs. Sequences of GAI were obtained for 190 'Herbie' genotypes.

### Phenotypic data analysis

For 'Herbie' and Cc separately, analyses of variance were conducted using GLM procedure (SS type 3) in SAS 8.1 [31]. Genotypic effect for each trait in each experiment was analysed using the model:

(4) $$Y_{ij}=\mu +\;G_{i}+\;B_{j}+\;e_{ij},$$

with *Y_ij _*the value of genotype *i *taken in the block *j*, μ the population average, *G_i _*the effect of genotype *i*, *B_j _*the effect of the block *j *and e_ij _the residual. For each trait, the broad sense heritability (h^2^) was estimated as:

(5) $${\text{h}}^{\text{2}}\;=\;\frac{\sigma_{GE}^{2}\;}{\sigma_{GE}^{\text{2}}\;+\;\sigma_{\text{E}}^{\text{2}}}$$

Genotypic variance ($\sigma_{GE}^{2}$) and residual variance ($\sigma_{\text{E}}^{\text{2}}$) were estimated using the VARCOMP procedure of SAS with the method *residual maximum likelihood algorithm *REML [31].

For 'Herbie', a general analysis of variance taking into account the two periods of measurement (spring/autumn) was also performed. Plants used in spring and in autumn were independent clones, so the model used was:

(6) $$Y_{ijk}=\mu +\;G_{i}+\;P_{k}+\;{\left(G\;\times P\right)}_{ik}+\;B_{j}\left(P_{k}\right)\;+\;e_{ijk}$$

with *Y_ijk _*the value of genotype *i *taken in the block *j *for the period *k*, μ the average of the population, *G_i _*the effect of genotype *i *(random effect), Period_k _the effect of period *k *(fixed effect), (G × P)_ik _the effect of the interaction Genotype × Period (random effect), *B_j _(P_k_) *the effect of block *j *nested in period *k *(fixed effect) and e_ijk _the residual (random effect). *G *and *P *effects were tested using *G × P *interaction as residual and *G × P *interaction was tested using the model residuals.

For each trait, the broad sense heritability (h^2^) was estimated as:

(7) $${\text{h}}^{\text{2}}\;=\;\frac{\sigma_{GE}^{2}\;}{\sigma_{GE}^{\text{2}}\;{\mathrm{+\sigma}}_{GExPeriod}^{\text{2}}{\mathrm{+\sigma}}_{\text{E}}^{\text{2}}}$$

Genotypic variance ($\sigma_{GE}^{2}$), genotype × period interaction variance ($\sigma_{GExPeriod}^{2}$) and residual variance ($\sigma_{\text{E}}^{\text{2}}$) were estimated using the VARCOMP procedure of SAS with the method REML [31].

Pearson's correlations between traits were calculated on the adjusted means by genotype (option LSMEANS of GLM procedure) using the CORR procedure of SAS [31].

### Population substructure analysis

STRUCTURE software version 2.3 [5] was used to estimate the substructure of 'Herbie'. The length of the burn-in period was 50,000 and the number of MCMC replications after the burn-in was 100,000 for each. The given number of populations (K) was varied between 1 and 10. Computing was performed 50 times for each K value.

### GAI polymorphism analysis

The identity of amplified sequences was checked using a BLASTN on NCBI http://www.ncbi.nlm.nih.gov/BLAST/. The sequences were aligned and manually checked using STADEN package 1.6.0 http://staden.sourceforge.net/. Data were formatted using TRITIPOL http://bioweb.ensam.inra.fr/tritipol/ and REFSEQV5 (pers. com. Fabienne Granier, INRA, Versailles, France). Haplotype phase between SNPs was estimated using PHASE 2.1 software [39,40]. Haplotype number and haplotypic genotype for each individual were inferred.

### LD analysis

The r^2 ^values of gametic LD between SNPs were determined according to Hill and Robertson [41] on the haplotypes inferred by PHASE using DNAsp software [42]. Genotypic LD between each pair of SNPs or SSRs was calculated using GENEPOP software [43].

### Association study analysis

The association study was carried out independently for each period of measurement by using adjusted means per genotype. Three different approaches were used to test the association between the polymorphism of *GAI *and leaf elongation parameters:

1) a multiple linear regression analysis between leaf elongation parameters and SNPs. The stepwise method was used to fit a linear model of the general form:

(8) $$Y_{i}=\;\beta_{0}+\;\beta_{1}X_{1}+\;\beta_{2}X_{2}+\;\ldots \;+\;\beta_{n}X_{n}+\;\varepsilon_{i}$$

where *Yi *is any dependent variable (in our case: *Llength, LERmax or LED);X1, X2,..., Xn *are the independent variables (in our case: *20 SNPs*), *β0, β1, β2,..., βn*, the regression coefficients and εi the error term. It was computed with the STEPWISE option of REG procedure in SAS [31].

2) a Scheffe's multiple comparison analysis for orthogonal linear contrasts [44] in order to test the effect of presence versus absence of each not rare haplotype (i.e. more than 10 plants holding the haplotype). It was computed with the GLM procedure in SAS [31] following the model given by Eq. 4.

3) a tree-scanning analysis of the phenotypic data against the haplotype tree. It was performed with TREESCAN 1.0 http://darwin.uvigo.es/software/treescan.html[45,46]. This method allows testing the mean difference of a trait between two groups obtained for each branch of a phylogenetic tree. Haplotypes present more than 10 times in the population were used to construct a phylogenetic tree with PHYLIP 3.67 http://evolution.genetics.washington.edu/phylip.html using maximum parsimony. In the execution of the TREESCAN program the number of permutations was 5 000, and the minimum class size was set to five.

## Results

### Phenotypic analysis

*Llength*, *LERmax *and *LED *were determined for 216 plants of 'Herbie' in spring and autumn and on 100 plants of a perennial ryegrass core-collection (Cc) in autumn. Three replicates were used. Genetic effect was highly significant for all traits for both Herbie and the Cc and for both growing periods. Heritability was high for *Llength *and *LERmax *and medium for *LED *(Table 1). For all traits, a significant Genotype × Period interaction was detected in 'Herbie' and moderate heritabilities were found for both periods (Table 2). The variability of 'Herbie' was as high as the variability of the 'Cc' (Table 3). Correlation values between variables are presented in Table 4 for 'Herbie' including both spring and autumn measurements. For the 'Cc', correlations between variables during autumn were highly significant (*P *value < 0.001) between *Llength *and both *LERmax *(0.87) and *LED *(0.46), but not significant (*P *value > 0.05) between LERmax and LED. In both 'Herbie' and the 'Cc', leaf length was significantly correlated to both *LERmax *and *LED *with higher values for *LERmax*. On the other hand, *LERmax *and *LED *were not or were weakly correlated. Leaf parameters measured in spring were correlated to those measured in autumn with a stronger correlation for *Llength *and *LERmax *than for *LED*.

**Table 1:** Heritability of leaf parameters per period

Populations	Periods	Variables	σ^2^_E_	σ^2^_GE_	CV (%)	H^2^
**Herbie**		***Llength***	1176	4524	11	0.79
	**Spring**	***LED***	658	700	9	0.52
		***LERmax***	0.024	0.096	9	0.80
	
		***Llength***	1720	3668	13	0.68
	**Autumn**	***LED***	1233	1003	12	0.45
		***LERmax***	0.028	0.063	11	0.69

**Cc**		***Llength***	1634	4477	14	0.73
	**Autumn**	***LED***	1557	836	15	0.35
		***LERmax***	0.033	0.094	12	0.74

Analyses of variance on leaf length *(Llength)*, leaf elongation duration *(LED) *and maximum leaf elongation rate *(LERmax) *for spring and autumn on 'Herbie' and for autumn on the core collection (Cc). Are indicated: error (σ^2^E) and genotypic (σ^2^GE) variances, coefficient of variation (CV) and heritability (H^2^)

**Table 2:** Heritability of leaf parameters over periods

Variables	σ^2^_E_	σ^2^_GE_	σ^2^_Period_	σ^2^_GE × Period_	CV	H^2^
***Llength***	1430	19569	55510	4467	12	0.55
***LED***	928	4635	147004	1680	11	0.32
***LERmax***	0.03	0.37	18	0.09	10	0.53

Analyses of variance on leaf length (Llength), leaf elongation duration (LED) and maximum leaf elongation rate (LERmax) considering two periods (spring and autumn) on 'Herbie'. Are indicated: error (σ^2^E), genotypic (σ^2^GE), period (σ^2^Period) and genotype × period interaction (σ^2^GE × Period) variances, coefficient of variation (CV) and heritability (H^2^)

**Table 3:** Distributions of leaf parameters

Populations	Period	Variables	Means	Minimum	Maximum	CV in %
		***Llength***	316	97	509	22
	**Spring**	***LERmax***	1.74	0.88	2.65	18
**Herbie**		***LED***	274	165	355	11
	
		***Llength***	306	144	474	22
	**Autumn**	***LERmax***	1.51	0.84	2.56	18
		***LED***	295	198	448	13
		***Llength***	272	128	489	26

**Cc**	**Autumn**	***LERmax***	1.48	0.80	2.56	22
		***LED***	272	181	350	14

Distribution of individuals (means over three clones) for leaf length *(Llength)*, leaf elongation duration *(LED) *and maximum leaf elongation rate *(LERmax) *in spring and autumn for 'Herbie' and in autumn for the core collection (Cc). CV indicates the coefficient of variation

**Table 4:** Correlations of leaf parameters over two periods

			*Spring*			Autumn	
		
		*Llength*	*LERmax*	*LED*	*Llength*	*LERmax*	*LED*
	***Llength***						
***Spring***	***LERmax***	0.90 ***					
	***LED***	0.55 ***	0.20 **				

	***Llength***	0.64 ***	0.62 ***	0.25 ***			
**Autumn**	***LERmax***	0.51 ***	0.62 ***	0.01 NS	0.82 ***		
	***LED***	0.38 ***	0.18 *	0.47 ***	0.60 ***	0.02 NS	

Correlations between leaf length *(Llength)*, leaf elongation duration *(LED) *and maximum leaf elongation rate *(LERmax) *for two periods (spring and autumn) for 'Herbie'

NS: not significant

*** Significant at 0.001

** Significant at 0.01

* Significant at 0.05

### Population substructure analysis

Results from the analysis with STRUCTURE software, using one single sequence repeat (SSR) marker per linkage group, showed no evidence of substructure in 'Herbie' (Table 5). Furthermore, as expected between unlinked markers in a population presenting no substructure, no significant genotypic LD was detected between pairs of SSRs.

**Table 5:** Probability of the populations (K) number for K varying from 1 to 10 using structure

	Probability of K
**1**	1
**2**	10^-57^
**3**	10^-177^
**4**	10^-209^
**5**	10^-203^
**6**	10^-233^
**7**	10^-256^
**8**	10^-274^
**9**	10^-270^
**10**	10^-247^

### GAI polymorphism

Twenty SNPs were detected in the 370 bp GAI sequence of 'Herbie' (Table 6). This corresponds to an average of 12 bp between two consecutive SNPs. Among these SNPs, six presented rare alleles with frequencies lower than 10% and one (GAI206) was not synonymous. PHASE software revealed the existence of 39 haplotypes (Table 7). Nine of them were present in more than 10 genotypes.

**Table 6:** Characterisation of the 20 SNPs observed in the *GAI *sequence of 'Herbie'

**SNP no**.	SNP position	Polymorphism	Frequency
1	GAI006	A/C	0.01
2	GAI024	G/C	0.01
3	GAI039	A/G/C	0.20/0.19
4	GAI042	A/C	0.27
5	GAI045	T/C	0.21
6	GAI048	G/C	0.88
7	GAI051	A/G	0.59
8	GAI054	A/G	0.51
9	GAI060	G/C	0.85
10	GAI069	G/C	0.54
11	GAI072	G/C	0.08
12	GAI084	G/C	0.52
13	GAI099	G/C	0.42
14	GAI114	A/C	0.20
15	GAI138	G/C	0.76
16	GAI156	G/C	0.21
17	GAI189	G/C	0.85
18	GAI206	G/C	0.02
19	GAI222	A/G	0.94
20	GAI228	T/G	0.02

The frequencies refer to the first (or first two) allele of each locus presented in column Polymorphism

**Table 7:** Haplotypes of *GAI *in 'Herbie' and their number inferred using PHASE 2

**Haplotype no**.	Haplotype	Inferred no. in sample
1	CCAACGGAGGGGGCGCGCAG	1
**2**	**CCACTGAAGCCGGCGCGCAG**	**66**
3	CCACTGAAGCCGGCGGGCAG	1
4	CCACCGAGCGCCCCGCGCAG	1
5	CCACCGGAGGGGGCGCGCAG	3
6	CCGATGGGGCCGCCGCGCAG	1
7	CCGACGGAGGGGCCGCGCAG	1
8	CCGACGGGGCCGGCGCGCAG	3
**9**	**CCGACGGGGCCGGCGCCCAG**	**57**
**10**	**CCGACGGGGCCGCCGCGCAG**	**17**
11	CCGACGGGGCCGCAGCGCAG	3
12	CCGACGGGGCCCCCGCGCAG	1
13	CCGACGGGGGCCGCGCCCAG	1
14	CCGACGGGGGCCGCGGGCAG	2
**15**	**CCGACCAGGCCGGCGCGCAG**	**14**
16	CCGACCGGGCCCCCCCGCAG	1
17	CCGCTGAAGCCCGCGCGCAG	1
18	CCGCTGAACGCCGCGCGCAG	3
19	CCGCTGGAGCCCGCGCGCAG	1
20	CCGCTGGGGCCGGCGCGCAG	1
21	CCGCCGAAGGCCCCGCGCAG	3
22	CCGCCGAAGGCCCCCCGGGT	8
23	CCGCCGAAGGCCCACCGCAG	1
**24**	**CCGCCGAAGGCCCACGGCAG**	**56**
**25**	**CCGCCGAAGGCCCACGGCGG**	**15**
26	CCGCCGAGCGCGCCGCGCAG	1
**27**	**CCGCCGAGCGCCCCGCGCAG**	**50**
28	CCGCCGGAGGCCCCGCGCAG	2
29	CCGCCGGAGGGGGCGCGCAG	1
**30**	**CCGCCGGAGGGGCCGCGCAG**	**18**
31	CCGCCGGAGGGGCCCCGCAG	5
32	CCGCCGGAGGGCCCGCGCAG	1
33	CCGCCGGACGCCCCCGGCAG	3
34	CCGCCGGGGGCCCCGCGCAG	3
35	CCGCCGGGGGCCCCGGGCAG	1
36	CCGCCCGGGCCCCCCCGCAG	3
**37**	**CCGCCCGGGGCCCCGCGCAG**	**26**
38	CGGCCCGAGGCGCCCGGCAG	1
39	ACACTGAAGCCGGCGCGCAG	3

The nine most abundant haplotypes are indicated in bold. In these haplotypes, SNP069 is underlined

### LD analysis

**Figure 4:**
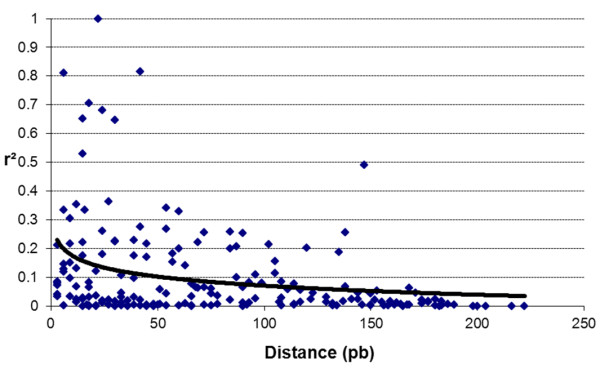
**Pattern of gametic linkage disequilibrium (LD) decays**. LD was measured between SNPs pairs, with r^2^, from haplotypic data of GAI gene inferred by PHASE software. A logarithm fitting-curve is presented in black (R^2 ^= 0.09).

Gametic LD decreased rapidly with the distance (Figure 4). Indeed, r^2 ^values became lower than 0.1 beyond 150 bp. Genotypic LD results were in agreement with Gametic LD results (data not shown). For distances lower than 150 bp, more than 35% of SNP pairs presented a significant LD (threshold at 0.01 after a Bonferroni correction). Nonetheless, beyond 150 bp, SNP pairs did not present any significant deviation from linkage equilibrium.

### Association study between GAI and leaf growth parameters

Three statistical methods were used to detect the association between the phenotypic polymorphism and GAI polymorphism.

The first method was a stepwise regression between leaf parameters and the 20 SNPs of GAI found in 'Herbie' (Eq. 8). The results are summarized in Table 8. It showed that, depending on the leaf parameter, three to six SNPs explained 8-14% of the phenotypic variance (R^2^). One SNP, named SNP069, appeared to be particularly interesting since it explained 2-5% of each parameter variance in both spring and autumn. It explained a difference of leaf length equal to 42 mm, for average leaf length of 312 mm, in spring and a difference equal to 30 mm, for an average of 303 mm, in autumn. It is noteworthy that the highest values of leaf growth parameters were obtained for heterozygous individuals showing a superdominance effect at SNP069.

**Table 8:** Association between GAI and leaf parameters polymorphisms: method 1

Periods	Variables	SNP	Pr > F^a^	Partial R^2 ^in %	Global R^2 ^in %	Average value	Effect of genotypes compared to the average		
							1/1*	2/2	1/2
	***Llength***	**SNP069**	0.0038	5	9	312	5	-27	15
	**(mm)**	SNP060	0.0361	3			7	-59	-15
		SNP039	0.0779	2			41	-21	15
**Spring**	***LERmax***	**SNP069**	0.0094	4	10	1.732	0.038	-0.122	0.059
	**(mm°C^-1^d^-1^)**	SNP099	0.0463	2			0.063	-0.067	0.026
		SNP206	0.0734	2			-0.007	/	0.162
		SNP189	0.0727	2			-0.011	-0.179	0.064
	***LED***	**SNP069**	0.0783	2	8	273	1	-6	9
	**(°Cd)**	SNP060	0.0969	2			2	0	-8
		SNP039	0.1004	2			13	-5	8
		SNP048	0.1441	1			-1	-9	2

	***Llength***	**SNP069**	0.0121	4	11	303	-6	-17	13
	**(mm)**	SNP099	0.025	3			3	-6	4
		SNP048	0.0361	3			-5	-48	23
		SNP222	0.074	2			-2		16
**Autumn**	***LERmax***	**SNP069**	0.0064	4	14	1.507	0.081	-0.019	0.047
	**(mm°C^-1^d^-1^)**	SNP048	0.0555	2			-0.019	-0.096	0.089
		SNP114	0.0799	2			0.012	-0.508	-0.003
		SNP156	0.0838	2			0.01	-0.116	-0.005
		SNP099	0.1454	1			0.038	-0.024	0.004
		SNP189	0.0448	2			0	-0.061	0.039
	***LED***	SNP051	0.0176	3	8	297	9	1	-6
	**(°Cd)**	**SNP069**	0.0512	2			-5	-6	5
		SNP114	0.1167	1			-4	-23	8
		SNP048	0.1362	1			-1	-33	6

Results of stepwise regressions for leaf length *(Llength*), leaf elongation rate *(LERmax) *and leaf elongation duration *(LED) *in spring and autumn on 'Herbie'. Are indicated: significant SNPs (*P*-value 0.15), the part of the phenotypic variation explained by each SNP (partial R^2^), the part of the phenotypic variation explained by all SNPs (global R^2^), the average value of each trait and the genotype effect compared to the average

* 1/1 and 2/2 indicate the two homozygous classes and 1/2 indicates the heterozygous class

^a ^Pr > F is the is the probability of having a tabulated F value at least as extreme as the calculated one

The second method consisted of a Scheffé analyses in order to test the effect of presence *versus *absence of the different haplotypes on each trait (Table 9). The comparisons of means for the significant contrasts are presented in Table 10.

**Table 9:** Association between GAI and leaf parameters polymorphisms: method 2

Haplo-types	Sample size +	Sample size -	Leaf length		LERmax		Leaf Elongation Duration	
			**spring**	**autumn**	**spring**	**autumn**	**spring**	**autumn**

2	60	126	**0.042**	0.934	0.038	0.822	0.713	0.286
9	50	136	0.164	0.807	0.118	0.220	0.815	0.164
10	17	169	0.858	0.521	0.701	0.488	0.582	0.058
15	14	172	0.533	0.109	0.115	0.039	0.449	0.472
24	54	132	**0.012**	**0.002**	**0.004**	**0.001**	0.083	0.842
25	15	171	0.850	0.089	0.423	0.979	0.310	**0.006**
27	45	141	0.098	0.115	0.384	0.415	0.167	0.427
30	17	169	0.175	**0.018**	0.808	0.207	**0.001**	**0.023**
37	25	161	0.801	0.151	0.937	0.321	0.488	0.211

*P*-values (α) of Scheffé analyses for the effect of presence (+) versus absence (-) of a haplotype for leaf length (Llength), maximum leaf elongation rate (LERmax) and leaf elongation duration (LED) in spring and autumn on 'Herbie'. The nine most abundant haplotypes were tested. The numbers of samples including the haplotype (+) or not (-) are indicated. The values below 0.05 are in bold

**Table 10:** Means per class presence (+) versus absence (-) of a haplotype for significant variables in Table 9

Haplo-types		Leaf length (mm)		LERmax (mm/°Cd)		Leaf Elongation Duration (°Cd)	
		**spring**	**autumn**	**spring**	**autumn**	**spring**	**autumn**

2	**-**	305					
	**+**	329					
24	**-**	313	305	1.74	1.52		
	**+**	310	299	1.71	1.47		
25	**-**						296
	**+**						306
30	**-**		303			271	296
	**+**		307			286	300

**Figure 5:**
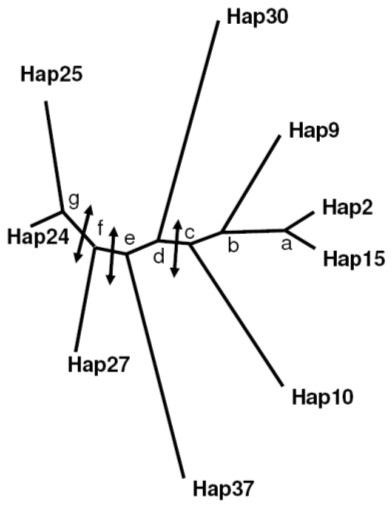
**Haplotype tree of the SNP polymorphism from the GAI gene**. The haplotypes are shown in Table 6. Only haplotypes present more than 10 times were taken into account. The arrows indicate the transitions with a Psim higher than 0.05 in the tree-scanning analysis. The tree was obtained from the program PhyloDraw (contact: jhchoi@pearl.cs.pusan.ac.kr) on the basis of the treefile output from PHYLIP.

The third method was a tree-scanning analysis of associations between haplotypes and leaf parameters. The haplotype tree is presented in Figure 5 and the results of the TREESCAN analysis is presented in Table 11. The strongest effect of phylogenetic groups on all traits was on *LER*max in spring between the group of haplotypes: 2, 9, 10 and 15, and the group of haplotypes: 24, 25, 27, 30, 37. These two groups can be separated by polymorphism at SNP069 (Table 7). This separation had also an effect on *Llength *in spring. An effect was found on *LER*max in spring and in autumn between the group of haplotypes 24 and 25, and the group including all the other haplotypes. These two groups can be separated by SNP114 and SNP138 which were in complete linkage disequilibrium when considering the nine most abundant haplotypes (Table 7). For all tests, the corrected permutational *P*-values after monotonicity were higher than 0.05.

**Table 11:** Association between *GAI *and leaf parameters polymorphisms: method 3

Traits	Branch	*F-statistics*	*Pvk*	*Psim*	*PMon*
Llength_spr	c-d	3.7	0.04	0.03	0.41
LER_spr	c-d	5.1	0.06	0.007	0.16
LER_spr	f-g	4.1	0.04	0.02	0.30
LER_spr	e-f	3.2	0.03	0.04	0.50
LER_aut	f-g	3.3	0.04	0.04	0.52

Tree-scanning analysis of associations between haplotypes and leaf parameters: leaf length (Llength), leaf elongation rate (LER) and leaf elongation duration (LED) in spring (spr) and autumn (aut) on 'Herbie'. Branches with *Psim *(permutational probability before correction for multiple testing) lower than 0.05 are shown. Pvk is the proportion of the trait variation explained by the partition. PMon is the corrected permutational *P*-value after monotonicity is enforced

## Discussion

### Phenotypic analyses

The phenotypic variability of 'Herbie' was very high and similar to the one observed in a core collection (Cc). This high variability of 'Herbie' was also observed on molecular data [8]. Such variability can be explained by the high number of parents (336) in the initial polycross. This diversity shows that selection should be efficient even within a variety.

High heritability values were observed for leaf length and maximum leaf elongation rate (*LERmax*) within each period of measurements. Lower heritabilities were observed when taking into account both spring and autumn periods. These results are in accordance with values from different studies in forage grasses [16,17,47-49]. The significant Genotype × Period interaction indicates that the response of genotypes to environmental conditions did not follow the same trajectories. This is reflected by the correlation values between spring and autumn leaf length or *LERmax *which were only moderately high (Table 4). As previously observed by Ghesquière [17], leaf elongation duration (*LED*) had a lower heritability. This could be partly explained by a lack of precision in the *LED *estimates since no measurement could be performed at the beginning of leaf growth, during the hidden phase.

Spring and autumn leaf length averages in 'Herbie' were not significantly different. However, these similar leaf lengths were not reached by the same dynamics of leaf growth. It was observed a higher *LERmax *and a shorter *LED *in spring than in autumn, that could be explained by higher temperatures in spring (average of 16°C) than in autumn (average of 13°C).

The correlation between leaf length and *LERmax *was higher than the one between leaf length and *LED *in spring and in autumn for both 'Herbie' and the core collection. Similar results were observed by Ghesquière [17] in a collection of late heading perennial ryegrass ecotypes with correlations of 0.86 and 0.53 between adult leaf length and average leaf elongation rate and *LED*, respectively. It could be explained by the fact that at the beginning of leaf growing process there is a long period of slow growth with no significant effect on the final leaf length. However, depending on the plant material, the impact of *LERmax *and *LED *on final leaf length could differ greatly (unpublished data). Since these two traits appear poorly correlated, a given final leaf length can result from different combinations of a wide range of *LED *and *LER *values.

### GAI polymorphism

The density of SNPs was very high but comparable to that obtained in other genes in perennial ryegrass, bearing in mind that this parameter is highly variable over the genome [50,51]. Nevertheless, the number of haplotypes observed in 'Herbie' is relatively weak compared to the haplotype numbers expected. Knowing that there were 336 individuals in the initial polycross of the 'Herbie' variety and considering that they were unrelated, 672 haplotypes were expected under the assumption of total absence of LD. However, only 39 haplotypes were observed, and among those, 30 had a frequency lower than 2%. This implies that the parents of 'Herbie' were related and some haplotypes were highly selected.

### Substructure and LD analyses

Based on the analysis of 216 plants, we confirmed that Herbie presents no substructure and that LD decay was very short in the GAI gene as reported by Auzanneau [8] based on the analysis of 47 plants. The absence of substructure and this rapid decline of LD with the genetic distance in Herbie were expected owing to the high number of parents and the variability in the initial polycross of the variety. Moreover, LD decline in perennial ryegrass is generally rapid and become not significant beyond about 1 kb due to its outbreeding reproductive system [51-53]. This rapid LD decline and the absence of substructure allowed a "candidate gene" approach in this genome region [4]

### Association study between GAI and leaf growth

This study in the highly diverse variety Herbie revealed that the GAI gene had a significant effect on leaf growth parameters: leaf length, maximum leaf elongation rate and leaf elongation duration, in both spring and autumn growing periods. This gene co-localized with QTL of lamina length found in spring and with QTL of leaf length and LER found in winter on linkage group 4 [28]. One SNP, SNP069, explained a part of the variability of all leaf length parameters in both spring and autumn. It was also found significant in the tree-scanning analysis. However this SNP does not induce an amino acid variation. Considering the rapid LD decline, the mutation responsible for leaf growth variability in 'Herbie' should be very close and highly linked to SNP069. In Arabidopsis, wheat and maize, mutants responsible for dwarf phenotypes present a deletion on the DELLA domain [19,21]. An association study on the whole gene sequence would be of interest to find the causal mutation. The possible association between the variation of the SNP069 and the variation of leaf length in 'Herbie' only by chance without any physical link between the SNP069 and the causal mutation (false positive) can be considered. This would imply that the SNP069 and a causal mutation elsewhere on the genome were in linkage disequilibrium. This LD can't come from a population substructure as we demonstrated the absence of structure in the population. It could come from a drift due to the sampling of a limited number of individuals (216) but this seems unlikely regarding the high significance of the SNP069 on leaf length. Nevertheless, in order to test the real physical association between the SNP069 and a causal mutation for leaf length, it would be interesting to create populations with GG or CC at the SNP069 and to compare their leaf length.

A superdominance effect was found for all leaf growth parameters on SNP069 except for *LERmax *in autumn. This could be due to two very close SNPs with dominant effects with the favourable alleles in repulsion. This superdominance effect could also be explained by a real complementary effect of both alleles at a single SNP. Whatever the origin of the superdominance effect, this observation should have consequences on breeding strategies. The objective of molecular assisted selection could thus be to associate two alleles instead of fixing favourable alleles.

The results of the three methods used for the association study were all in agreement. Nevertheless, the maximum number of associations was found with the regression analysis using SNPs which seems more effective since it explained a higher level of phenotypic variance than the other two methods. A possible explanation is that haplotypes cumulate alleles of several SNPs which could have opposite effects leading to a decrease in the difference between haplotypes.

In the present study a single panmictic population, a synthetic variety, allowed us to detect a strong association between a SNP polymorphism and a trait. In the study of Skot [53] a strong association was detected between one SNP:4443 of the LpHD1 gene and heading date (HD). Nine populations of perennial ryegrass, each including 96 plants, were used. Despite an overall association of SNP:4443 and HD, this association was more or less strong depending on the population. This shows that LD varies between populations leading to associations in some populations but not in others. Regarding this observation, it would be of interest to perform association studies within panmictic populations rather than to use individuals from several populations. Indeed, different associations can be found depending on the population used.

Unlike the findings of Thornsberry [54] in maize, we did not observe an effect of GAI gene on heading date in 'Herbie' (data not shown). However, we studied only a small part of the gene.

## Conclusions

In this study, we showed that i) association studies can be performed following the "candidate gene" approach on a synthetic variety with a wide genetic basis, and ii) the detection of association between phenotypic polymorphism and sequence polymorphism was more powerful using SNP polymorphism than haplotypic polymorphism. These observations could impact the way of using molecular information in plant breeding, in particular in outbreeding species. Indeed, the genetic basis of important traits could be directly detected in breeder populations and molecular markers used to increase favourable alleles.

## Abbreviations

GAI: Gibberellic acid insensitive; Llength: Leaf length; LER: Maximum leaf elongation rate; LED: Leaf elongation duration; SNP: Single nucleotide polymorphism; QTL: Quantitative trait locus; LD: Linkage disequilibrium; Cc: Core collection; Llength, Leaf length; SSR: Simple sequence repeat.

## Authors' contributions

JA phenotyped and genotyped the Herbie and Cc populations. AEG estimated LER and LED using a beta function. BJ and FG have been involved in the interpretation of data in genetics and ecophysiology, respectively. PB and CH coordinated the project and have made substantial contributions to conception and design of the project. PB, JA and AEG performed statistical analyses and wrote the manuscript. All the authors read and approved the final manuscript.

## References

[B1] Buckler ES Thornsberry JM Plant molecular diversity and applications to genomics Curr Opin Plant Biol 2002 5 107 111 10.1016/S1369-5266(02)00238-8 11856604

[B2] Flint-Garcia SA Thornsberry JM Buckler ES Structure of linkage disequilibrium in plants Annu Rev Plant Biol 2003 54 357 374 10.1146/annurev.arplant.54.031902.134907 14502995

[B3] Yu JM Buckler ES Genetic association mapping and genome organization of maize Curr Opin Biotechnol 2006 17 155 160 10.1016/j.copbio.2006.02.003 16504497

[B4] Rafalski A Application of single nucleotide polymorphisms in crop genetics Curr Opin Plant Biol 2002 54 94 100 10.1016/s1369-5266(02)00240-6 11856602

[B5] Pritchard JK Stephens M Rosenberg NA Donnelly P Association mapping in structured populations Am J Hum Genet 2000 67 170 181 10.1086/302959 10827107 PMC1287075

[B6] Thornsberry JM Goodman MM Oebley J Resovich S Ielsen D Buckler ES Dwarf8 polymorphisms associate with variation in flowering time Nat Genet 2001 28 286 289 10.1038/90135 11431702

[B7] Skot L Humphreys MO Armstead I Heywood S Skot KP Sanderson R Thomas ID Chorlton KH Hamilton NRS An association mapping approach to identify flowering time genes in natural populations of *Lolium perenne *(L.) Mol Breed 2005 15 233 245 10.1007/s11032-004-4824-9

[B8] Auzanneau J Huyghe C Julier B Barre P Linkage disequilibrium in synthetic varieties of perennial ryegrass Theor Appl Genet 2007 115 837 847 10.1007/s00122-007-0612-3 17701396

[B9] Wilkins P Humphreys M Progress in breeding perennial forage grasses for temperate agriculture J Agric Sci 2003 140 129 150 10.1017/S0021859603003058

[B10] Rhodes I The relationship between productivity and some components of canopy structure in ryegrass (*Lolium *spp.). I. Leaf length J Agric Sci 1969 73 315 319 10.1017/S0021859600019924

[B11] Rhodes I The relationship between productivity and some components of canopy structure in ryegrass (*Lolium* spp.). II. Yield, canopy structure and light interception J Agric Sci 1971 77 283 292 10.1017/S0021859600024436

[B12] Verdenal A De la simulation de la morphogénèse de l'appareil aérien du ray-grass anglais (Lolium perenne L.). Exploration d'un schéma cybernétique inspiré du concept d'auto-organisation et applications 2009 Poitiers (FRA): Université de Poitiers 207

[B13] Barre P Emile JC Betin M Surault F Ghesquière M Hazard L Morphological characteristics of perennial ryegrass leaves that influence short-term intake in dairy cows Agron J 2006 98 978 985 10.2134/agronj2005.0213

[B14] Hazard L Ghesquière M Evidence from the use of isozyme markers of competition in swards between short-leaved and long-leaved perennial ryegrass Grass Forage Sci 1995 50 241 248 10.1111/j.1365-2494.1995.tb02319.x

[B15] Cooper JP Edwards D The genetic control of leaf development in *Lolium*. I Assessment of genetic variation Heredity 1961 16 63 82 10.1038/hdy.1961.5

[B16] Rhodes I The relationship between productivity and some components of canopy structure in ryegrass (*Lolium* spp.) III. Spaced plant characters, their heritabilities and relationship to sward yield J Agric Sci 1973 80 171 176 10.1017/S002185960005718X

[B17] Ghesquière M Hazard L Betin M Breeding for management adaptation in perennial ryegrass (*Lolium perenne *L.). II. Genetic variability and heritability of leaf morphogenesis components Agronomie 1994 14 267 272 10.1051/agro:19940406

[B18] Ogawa M Kusano T Katsumi M Sano H Rice gibberellin-insensitive gene homolog, OsGAI encodes a nuclear-localized protein capable of gene activation at transcriptional level Gene 2000 245 21 29 10.1016/S0378-1119(00)00018-4 10713441

[B19] Peng JR Richards DE Hartley NM Murphy GP Devos KM Flintham JE Beales J Fish LJ Worland AJ Pelica F Sudhakar D Christou P Snape JW Gale MD Harberd NP 'Green revolution' genes encode mutant gibberellin response modulators Nature 1999 400 256 261 10.1038/22307 10421366

[B20] Koornneef M Elgersma A Hanhart CJ Van Loenen-Martinet EP Van Rijn L Zeevaart JAD A gibberellin insensitive mutant of *Arabidopsis thaliana* Physiol Plant 1985 65 33 39 10.1111/j.1399-3054.1985.tb02355.x

[B21] Peng JR Carol P Richards DE King KE Cowling RJ Murphy GP Harberd NP The Arabidopsis GAI gene defines a signaling pathway that negatively regulates gibberellin responses Genes Dev 1997 11 3194 3205 10.1101/gad.11.23.3194 9389651 PMC316750

[B22] Ikeda A Ueguchi-Tanaka M Sonoda Y Kitano H Koshioka M Futsuhara Y Matsuoka M Yamaguchi J Slender rice, a constitutive gibberelin response mutant, is caused by a null mutation of the SLR1 gene, an ortholog of the height-regulating gene GAI/RGA/RHT/D8 Plant Cell 2001 13 999 1010 11340177 10.1105/tpc.13.5.999 PMC135552

[B23] Foster C Slender: an accelerated extension growth mutant of barley Barley Genet Newsl 1977 7 24 27

[B24] Lanahan M Ho T Slender barley: a constitutive gibberellin-response mutant Planta 1988 175 107 114 10.1007/BF00402887 24221634

[B25] Jacobsen SE Olszewski NE Mutations at the spindly locus of Arabidopsis alter gibberellin signal-Transduction Plant Cell 1993 5 887 896 8400871 10.1105/tpc.5.8.887 PMC160324

[B26] Chardon F Virlon B Moreau L Falque M Joets J Decousset L Murigneux A Charcosset A Genetic architecture of flowering time in maize as inferred from quantitative trait loci meta-analysis and synteny conservation with the rice genome Genetics 2004 168 2169 2185 10.1534/genetics.104.032375 15611184 PMC1448716

[B27] Yamada T Forster JW QTL analysis and trait dissection in ryegrass (*Lolium *spp.) Humphreys MO 2005 Wageningen Academic Publishers 43 53

[B28] Barre P Moreau L Mi F Turner L Gastal F Julier B Ghesquiere M Quantitative trait loci for leaf length in perennial ryegrass (Lolium perenne L.) Grass Forage Sci 2009 64 310 321 10.1111/j.1365-2494.2009.00696.x

[B29] Verdenal A Combes D Escobar-Gutiérrez A A study of ryegrass architecture as a self-regulated system, using functional-structural plant modeling Funct Plant Biol 2008 35 911 924 10.1071/FP08050 32688842

[B30] Yin X Goudriaan J Antinga EA Os J Spiertz HJ A flexible sigmoid function of determination growth Ann Bot 2003 91 61 371 10.1093/aob/mcg029 PMC4244967 12547689

[B31] SAS Institute SAS language and procedure: usage. Version 8.1 2004 Cary, NC, USA: SAS Institute Inc

[B32] Escobar-Gutiérrez A Combes D Rakocevic M De Berranger C Eprinchard-Ciesla A Sinoquet H Varlet-Grancher C Functional relationships to estimate morphogenetically active radiation from PAR and solar broadband irradiance measurements Agric For Meteorol 2008 149 1244 1253

[B33] Lasseur B Lothier J Morvan-Bertrand A Escobar-Gutiérrez A Humphreys MO Prud'homme MP Impact of defoliation frequency on regrowth and carbohydrate metabolism in contrasting varieties of *Lolium perenne* Funct Plant Biol 2007 34 418 430 10.1071/FP06286 32689369

[B34] Saghai-Maroof MA Soliman K Jorgensen RA Allard RW Ribosomal DNA spacer-length polymorphisms in barley: mendelian inheritance, chromosomal location, and population dynamics PNAS 1984 81 8014 8018 10.1073/pnas.81.24.8014 6096873 PMC392284

[B35] Weising K Beyermann B Ramser J Kahl G Plant DNA fingerprinting with radioactive and digoxignated oligonucleotide probes complementary to simple repetitive DNA sequences Electrophoresis 1991 12 159 169 10.1002/elps.1150120211 2040264

[B36] Studer B Asp T Frei U Hentrup S Meally H Guillard A Barth S Muylle H Roldan-Ruiz I Barre P Koning-Boucoiran C Uenk-Stunnenberg G Dolstra O Skot L Skot KP Turner LB Humphreys MO Kolliker R Roulund N Nielsen KK Lubberstedt T Expressed sequence tag-derived microsatellite markers of perennial ryegrass (Lolium perenne L.) Mol Breed 2008 21 533 548 10.1007/s11032-007-9148-0

[B37] Faville M Vecchies A Schreiber M Drayton M Hughes L Jones E Guthridge K Smith K Sawbridge T Spangenberg G Bryan G Forster J Functionally associated molecular genetic marker map construction in perennial ryegrass (*Lolium perenne *L.) Theor Appl Genet 2004 110 12 32 10.1007/s00122-004-1785-7 15526086

[B38] Bolle C The role of GRAS proteins in plant signal transduction and development Planta 2004 218 683 692 10.1007/s00425-004-1203-z 14760535

[B39] Stephens M Donnelly P A comparison of Bayesian methods for haplotype reconstruction from population genotype data Am J Hum Genet 2003 73 1162 1169 10.1086/379378 14574645 PMC1180495

[B40] Stephens M Smith NJ Donnelly P A new statistical method for haplotype reconstruction from population data Am J Hum Genet 2001 68 978 989 10.1086/319501 11254454 PMC1275651

[B41] Hill WG Robertson A Linkage disequilibrium in finite populations Theor Appl Genet 1968 38 226 231 10.1007/BF01245622 24442307

[B42] Rozas J Sanchez-DelBarrio JC Messeguer X Rozas R DnaSP, DNA polymorphism analyses by the coalescent and other methods Bioinformatics 2003 19 2496 2497 10.1093/bioinformatics/btg359 14668244

[B43] Raymond M Rousset F GENEPOP (Version1.2): population genetics software for exact tests and ecumenicism J Hered 1995 86 248 249

[B44] Steel RGD Torrie JH Principles and procedures of statistics: a biometrical approach 1981

[B45] Posada D Maxwell TJ Templeton AR TreeScan: a bioinformatic application to search for genotype/phenotype associations using haplotype trees Bioinformatics 2005 21 2130 2132 10.1093/bioinformatics/bti293 15681571

[B46] Templeton AR Maxwell T Posada D Stengard JH Boerwinkle E Sing CF Tree scanning: a method for using haplotype trees in phenotype/genotype association studies Genetics 2005 169 441 453 15371364 10.1534/genetics.104.030080 PMC1448891

[B47] Edwards D Cooper JP The genetic control of leaf development in *Lolium*. II Response to selection Heredity 1963 18 307 317 10.1038/hdy.1963.32

[B48] Yamada T Jones ES Cogan NOI Vecchies AC Nomura T Hisano H Shimamoto Y Smith KF Hayward MD Forster JW QTL analysis of morphological, developmental, and winter hardiness-associated traits in perennial ryegrass Crop Sci 2004 44 925 935 10.2135/cropsci2004.0925

[B49] Horst GL Nelson CJ Asay KH Relationship of leaf elongation to forage yield of tall fescue genotypes Crop Sci 1978 18 715 719 10.2135/cropsci1978.0011183X001800050005x

[B50] Cogan N Ponting R Vecchies A Drayton M George J Dracatos P Dobrowolski M Sawbridge TI Smith K Spangenberg G Forster J Gene-associated single nucleotide polymorphism discovery in perennial ryegrass (*Lolium perenne *L.) Mol Genet Genomics 2006 276 101 112 10.1007/s00438-006-0126-8 16708235

[B51] Xing Y Frei U Schejbel B Asp T Lübberstedt T Nucleotide diversity and linkage disequilibrium in 11 expressed resistance candidate genes in *Lolium perenne* BMC Plant Biol 2007 7 43 10.1186/1471-2229-7-43 17683574 PMC1978496

[B52] Ponting RC Drayton MC Cogan NOI Dobrowolski MP Spangenberg GC Smith KF Forster JW SNP discovery, validation, haplotype structure and linkage disequilibrium in full-length herbage nutritive quality genes of perennial ryegrass (*Lolium perenne *L.) Mol Genet Genomics 2007 278 585 597 10.1007/s00438-007-0275-4 17647019

[B53] Skot L Humphreys J Humphreys MO Thorogood D Gallagher J Sanderson R Armstead IP Thomas ID Association of candidate genes with flowering time and water-soluble carbohydrate content in *Lolium perenne *(L.) Genetics 2007 177 535 547 10.1534/genetics.107.071522 17660575 PMC2013705

[B54] Thornsberry J Goodman M Doebley J Kresovich S Nielsen D Buckler E Dwarf8 polymorphisms associate with variation in flowering time Nat Genet 2001 28 286 289 10.1038/90135 11431702

